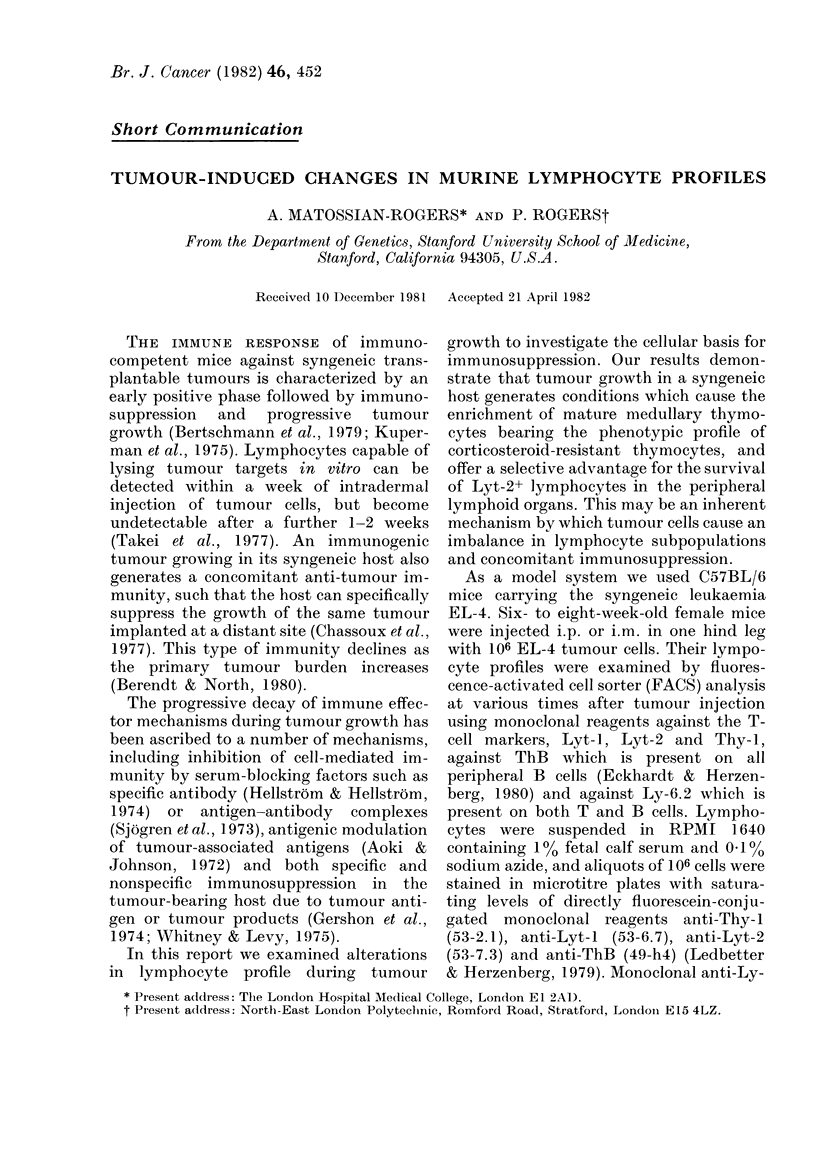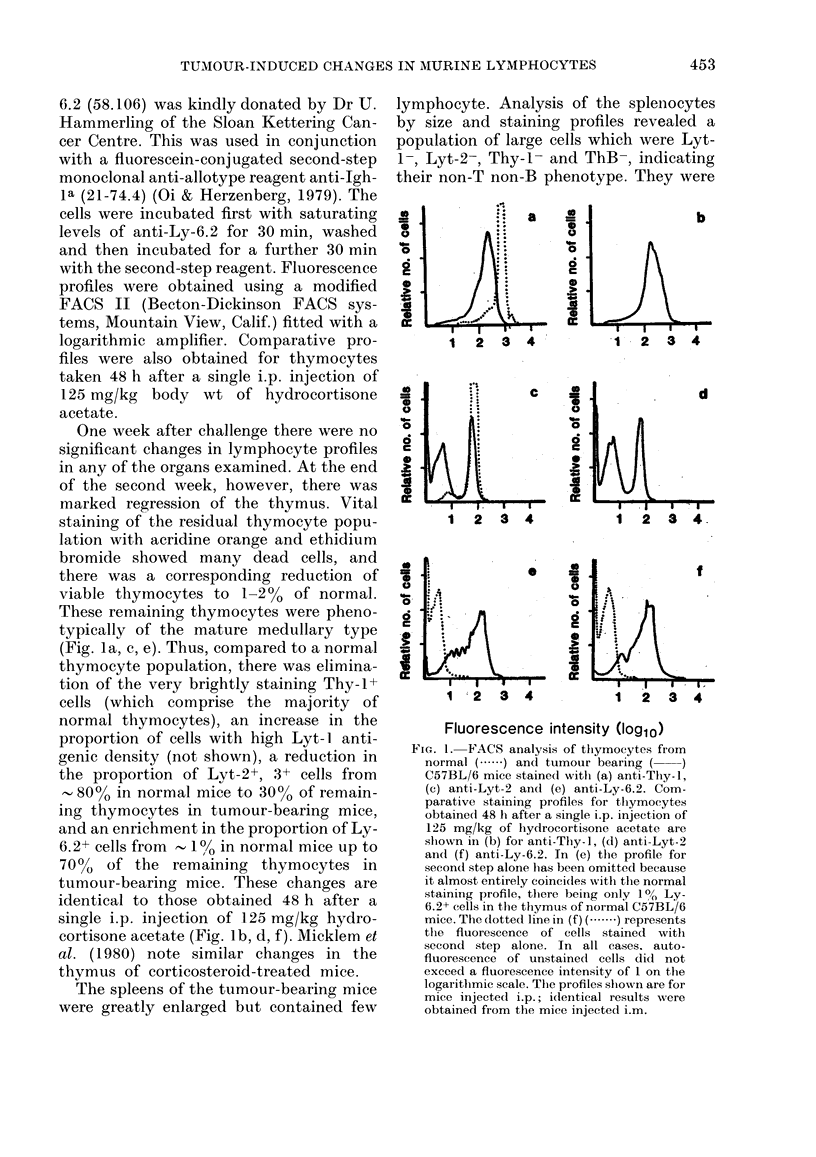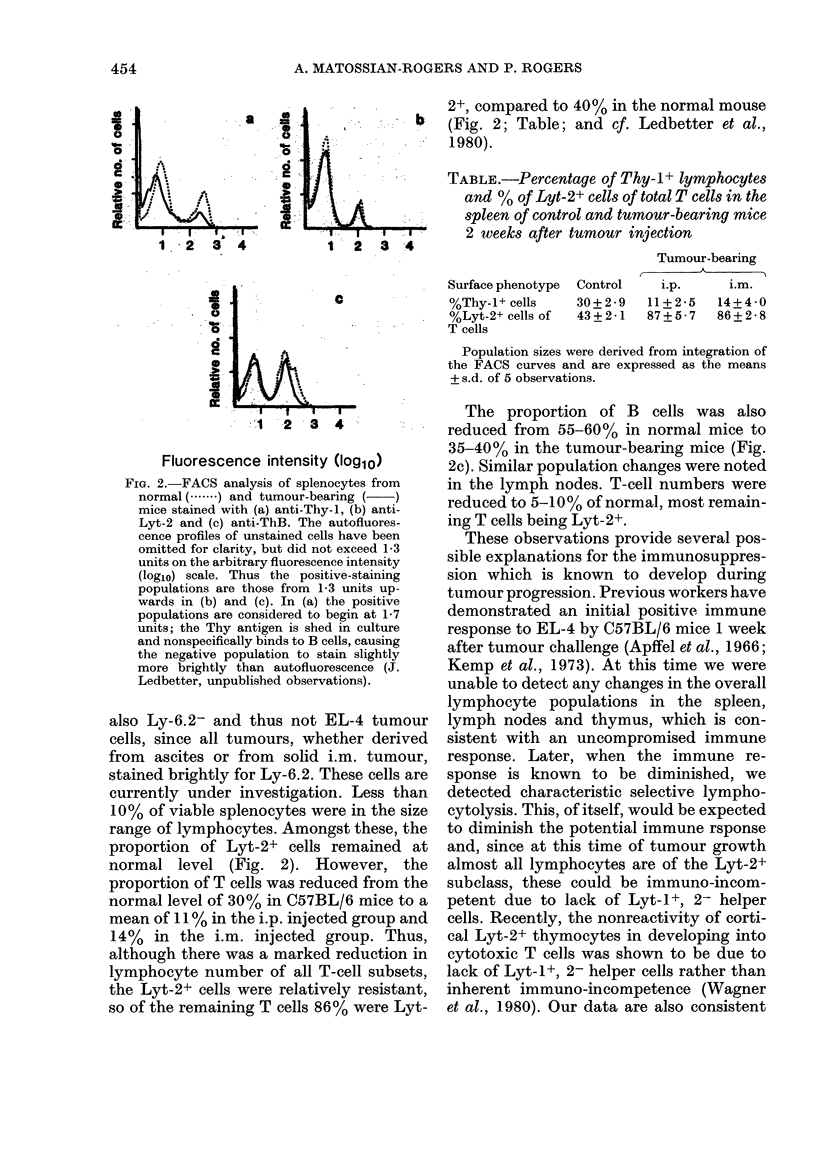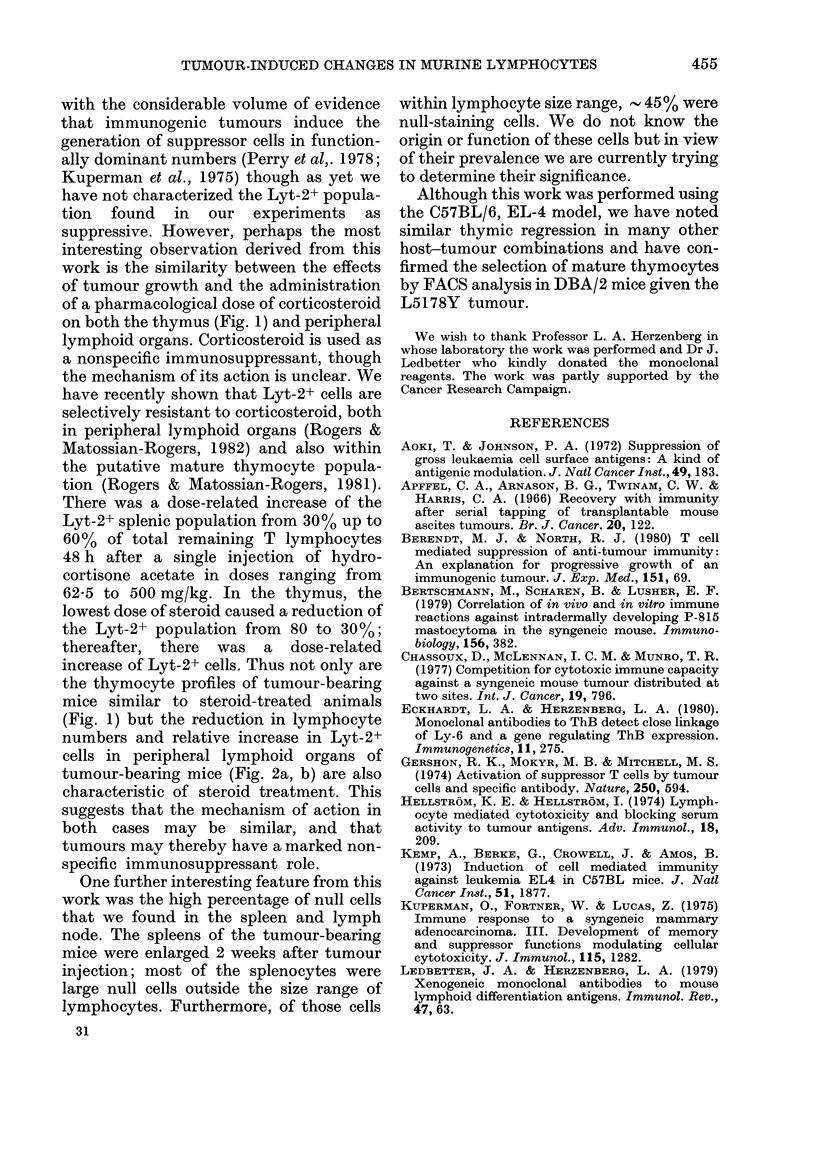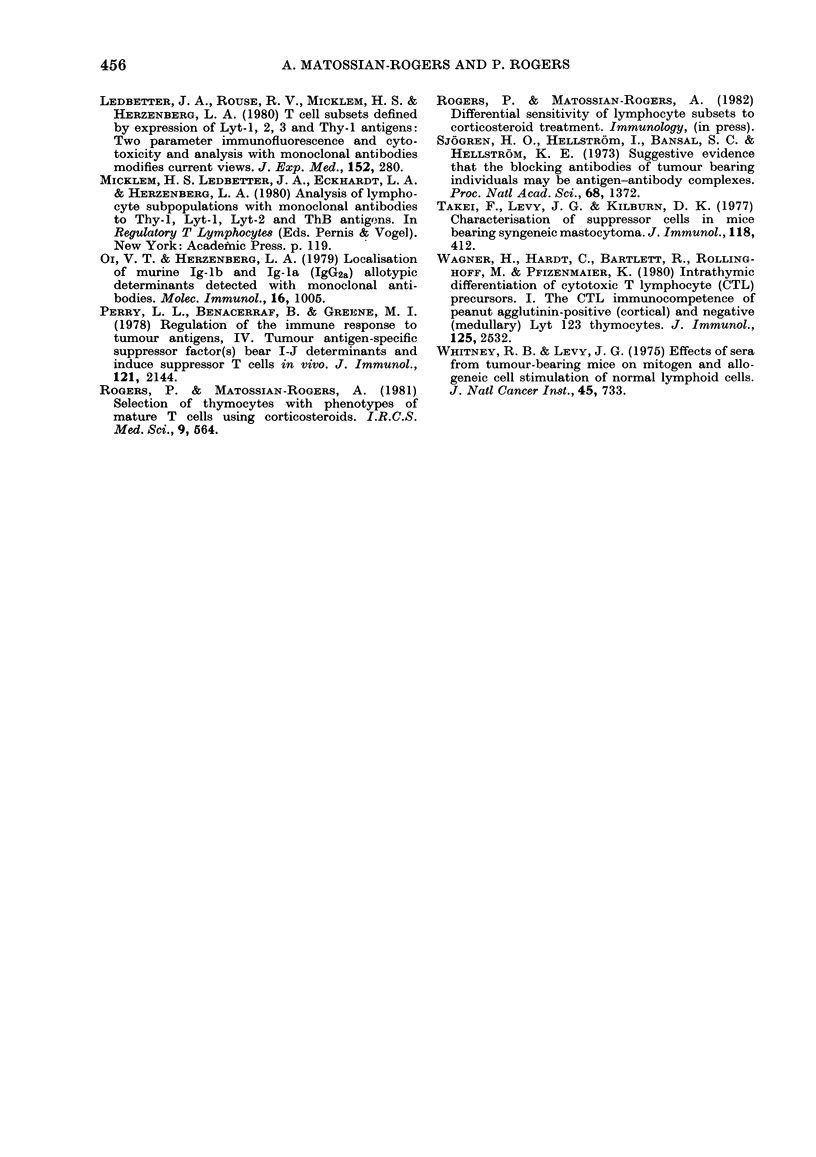# Tumour-induced changes in murine lymphocyte profiles.

**DOI:** 10.1038/bjc.1982.225

**Published:** 1982-09

**Authors:** A. Matossian-Rogers, P. Rogers


Br. J. Cancer (1982) 46, 452

### Short Communication

### TUMOUR-INDUCED CHANGES IN MURINE LYMPHOCYTE PROFILES

A. MATOSSIAN-ROGERS* AND P. ROGERS†

*From the Department of Genetics, Stanford University School of Medicine, Stanford, California 94305, U.S.A.*

Received 10 December 1981 Accepted 21 April 1982

THE IMMUNE RESPONSE of immunocompetent mice against syngeneic transplantable tumours is characterized by an early positive phase followed by immunosuppression and progressive tumour growth (Bertschmann *et al.*, 1979; Kuperman *et al.*, 1975). Lymphocytes capable of lysing tumour targets *in vitro* can be detected within a week of intradermal injection of tumour cells, but become undetectable after a further 1–2 weeks (Takei *et al.*, 1977). An immunogenic tumour growing in its syngeneic host also generates a concomitant anti-tumour immunity, such that the host can specifically suppress the growth of the same tumour implanted at a distant site (Chassoux *et al.*, 1977). This type of immunity declines as the primary tumour burden increases (Berendt & North, 1980).

The progressive decay of immune effector mechanisms during tumour growth has been ascribed to a number of mechanisms, including inhibition of cell-mediated immunity by serum-blocking factors such as specific antibody (Hellström & Hellström, 1974) or antigen–antibody complexes (Sjögren *et al.*, 1973), antigenic modulation of tumour-associated antigens (Aoki & Johnson, 1972) and both specific and nonspecific immunosuppression in the tumour-bearing host due to tumour antigen or tumour products (Gershon *et al.*, 1974; Whitney & Levy, 1975).

In this report we examined alterations in lymphocyte profile during tumour growth to investigate the cellular basis for immunosuppression. Our results demonstrate that tumour growth in a syngeneic host generates conditions which cause the enrichment of mature medullary thymocytes bearing the phenotypic profile of corticosteroid-resistant thymocytes, and offer a selective advantage for the survival of Lyt-2$^{+}$ lymphocytes in the peripheral lymphoid organs. This may be an inherent mechanism by which tumour cells cause an imbalance in lymphocyte subpopulations and concomitant immunosuppression.

As a model system we used C57BL/6 mice carrying the syngeneic leukaemia EL-4. Six- to eight-week-old female mice were injected i.p. or i.m. in one hind leg with $10^6$ EL-4 tumour cells. Their lympocyte profiles were examined by fluorescence-activated cell sorter (FACS) analysis at various times after tumour injection using monoclonal reagents against the T-cell markers, Lyt-1, Lyt-2 and Thy-1, against ThB which is present on all peripheral B cells (Eckhardt & Herzenberg, 1980) and against Ly-6.2 which is present on both T and B cells. Lymphocytes were suspended in RPMI 1640 containing 1% fetal calf serum and 0·1% sodium azide, and aliquots of $10^6$ cells were stained in microtitre plates with saturating levels of directly fluorescein-conjugated monoclonal reagents anti-Thy-1 (53-2.1), anti-Lyt-1 (53-6.7), anti-Lyt-2 (53-7.3) and anti-ThB (49-h4) (Ledbetter & Herzenberg, 1979). Monoclonal anti-Ly-

* Present address: The London Hospital Medical College, London E1 2AD.

† Present address: North-East London Polytechnic, Romford Road, Stratford, London E15 4LZ.

6.2 (58.106) was kindly donated by Dr U. Hammerling of the Sloan Kettering Cancer Centre. This was used in conjunction with a fluorescein-conjugated second-step monoclonal anti-allotype reagent anti-Igh-1$^a$ (21-74.4) (Oi & Herzenberg, 1979). The cells were incubated first with saturating levels of anti-Ly-6.2 for 30 min, washed and then incubated for a further 30 min with the second-step reagent. Fluorescence profiles were obtained using a modified FACS II (Becton-Dickinson FACS systems, Mountain View, Calif.) fitted with a logarithmic amplifier. Comparative profiles were also obtained for thymocytes taken 48 h after a single i.p. injection of 125 mg/kg body wt of hydrocortisone acetate.

One week after challenge there were no significant changes in lymphocyte profiles in any of the organs examined. At the end of the second week, however, there was marked regression of the thymus. Vital staining of the residual thymocyte population with acridine orange and ethidium bromide showed many dead cells, and there was a corresponding reduction of viable thymocytes to 1–2% of normal. These remaining thymocytes were phenotypically of the mature medullary type (Fig. 1a, c, e). Thus, compared to a normal thymocyte population, there was elimination of the very brightly staining Thy-1$^+$ cells (which comprise the majority of normal thymocytes), an increase in the proportion of cells with high Lyt-1 antigenic density (not shown), a reduction in the proportion of Lyt-2$^+$, 3$^+$ cells from ~80% in normal mice to 30% of remaining thymocytes in tumour-bearing mice, and an enrichment in the proportion of Ly-6.2$^+$ cells from ~1% in normal mice up to 70% of the remaining thymocytes in tumour-bearing mice. These changes are identical to those obtained 48 h after a single i.p. injection of 125 mg/kg hydrocortisone acetate (Fig. 1b, d, f). Micklem *et al.* (1980) note similar changes in the thymus of corticosteroid-treated mice.

The spleens of the tumour-bearing mice were greatly enlarged but contained few lymphocyte. Analysis of the splenocytes by size and staining profiles revealed a population of large cells which were Lyt-1$^-$, Lyt-2$^-$, Thy-1$^-$ and ThB$^-$, indicating their non-T non-B phenotype. They were

FIG. 1.—FACS analysis of thymocytes from normal (······) and tumour bearing (——) C57BL/6 mice stained with (a) anti-Thy-1, (c) anti-Lyt-2 and (e) anti-Ly-6.2. Comparative staining profiles for thymocytes obtained 48 h after a single i.p. injection of 125 mg/kg of hydrocortisone acetate are shown in (b) for anti-Thy-1, (d) anti-Lyt-2 and (f) anti-Ly-6.2. In (e) the profile for second step alone has been omitted because it almost entirely coincides with the normal staining profile, there being only 1% Ly-6.2$^+$ cells in the thymus of normal C57BL/6 mice. The dotted line in (f) (·······) represents the fluorescence of cells stained with second step alone. In all cases, autofluorescence of unstained cells did not exceed a fluorescence intensity of 1 on the logarithmic scale. The profiles shown are for mice injected i.p.; identical results were obtained from the mice injected i.m.

FIG. 2.—FACS analysis of splenocytes from normal (·······) and tumour-bearing (———) mice stained with (a) anti-Thy-1, (b) anti-Lyt-2 and (c) anti-ThB. The autofluorescence profiles of unstained cells have been omitted for clarity, but did not exceed 1·3 units on the arbitrary fluorescence intensity ($\log_{10}$) scale. Thus the positive-staining populations are those from 1·3 units upwards in (b) and (c). In (a) the positive populations are considered to begin at 1·7 units; the Thy antigen is shed in culture and nonspecifically binds to B cells, causing the negative population to stain slightly more brightly than autofluorescence (J. Ledbetter, unpublished observations).

also Ly-6.2$^-$ and thus not EL-4 tumour cells, since all tumours, whether derived from ascites or from solid i.m. tumour, stained brightly for Ly-6.2. These cells are currently under investigation. Less than 10% of viable splenocytes were in the size range of lymphocytes. Amongst these, the proportion of Lyt-2$^+$ cells remained at normal level (Fig. 2). However, the proportion of T cells was reduced from the normal level of 30% in C57BL/6 mice to a mean of 11% in the i.p. injected group and 14% in the i.m. injected group. Thus, although there was a marked reduction in lymphocyte number of all T-cell subsets, the Lyt-2$^+$ cells were relatively resistant, so of the remaining T cells 86% were Lyt-2$^+$, compared to 40% in the normal mouse (Fig. 2; Table; and *cf.* Ledbetter *et al.*, 1980).

TABLE.—*Percentage of Thy-*1$^+$ *lymphocytes and % of Lyt-*2$^+$ *cells of total T cells in the spleen of control and tumour-bearing mice 2 weeks after tumour injection*

| | | Tumour-bearing | |
|---|---|---|---|
| Surface phenotype | Control | i.p. | i.m. |
| %Thy-1$^+$ cells | 30±2·9 | 11±2·5 | 14±4·0 |
| %Lyt-2$^+$ cells of T cells | 43±2·1 | 87±5·7 | 86±2·8 |

Population sizes were derived from integration of the FACS curves and are expressed as the means ±s.d. of 5 observations.

The proportion of B cells was also reduced from 55–60% in normal mice to 35–40% in the tumour-bearing mice (Fig. 2c). Similar population changes were noted in the lymph nodes. T-cell numbers were reduced to 5–10% of normal, most remaining T cells being Lyt-2$^+$.

These observations provide several possible explanations for the immunosuppression which is known to develop during tumour progression. Previous workers have demonstrated an initial positive immune response to EL-4 by C57BL/6 mice 1 week after tumour challenge (Apffel *et al.*, 1966; Kemp *et al.*, 1973). At this time we were unable to detect any changes in the overall lymphocyte populations in the spleen, lymph nodes and thymus, which is consistent with an uncompromised immune response. Later, when the immune response is known to be diminished, we detected characteristic selective lymphocytolysis. This, of itself, would be expected to diminish the potential immune rsponse and, since at this time of tumour growth almost all lymphocytes are of the Lyt-2$^+$ subclass, these could be immuno-incompetent due to lack of Lyt-1$^+$, 2$^-$ helper cells. Recently, the nonreactivity of cortical Lyt-2$^+$ thymocytes in developing into cytotoxic T cells was shown to be due to lack of Lyt-1$^+$, 2$^-$ helper cells rather than inherent immuno-incompetence (Wagner *et al.*, 1980). Our data are also consistent with the considerable volume of evidence that immunogenic tumours induce the generation of suppressor cells in functionally dominant numbers (Perry *et al*,. 1978; Kuperman *et al.*, 1975) though as yet we have not characterized the Lyt-2$^+$ population found in our experiments as suppressive. However, perhaps the most interesting observation derived from this work is the similarity between the effects of tumour growth and the administration of a pharmacological dose of corticosteroid on both the thymus (Fig. 1) and peripheral lymphoid organs. Corticosteroid is used as a nonspecific immunosuppressant, though the mechanism of its action is unclear. We have recently shown that Lyt-2$^+$ cells are selectively resistant to corticosteroid, both in peripheral lymphoid organs (Rogers & Matossian-Rogers, 1982) and also within the putative mature thymocyte population (Rogers & Matossian-Rogers, 1981). There was a dose-related increase of the Lyt-2$^+$ splenic population from 30% up to 60% of total remaining T lymphocytes 48 h after a single injection of hydrocortisone acetate in doses ranging from 62·5 to 500 mg/kg. In the thymus, the lowest dose of steroid caused a reduction of the Lyt-2$^+$ population from 80 to 30%; thereafter, there was a dose-related increase of Lyt-2$^+$ cells. Thus not only are the thymocyte profiles of tumour-bearing mice similar to steroid-treated animals (Fig. 1) but the reduction in lymphocyte numbers and relative increase in Lyt-2$^+$ cells in peripheral lymphoid organs of tumour-bearing mice (Fig. 2a, b) are also characteristic of steroid treatment. This suggests that the mechanism of action in both cases may be similar, and that tumours may thereby have a marked nonspecific immunosuppressant role.

One further interesting feature from this work was the high percentage of null cells that we found in the spleen and lymph node. The spleens of the tumour-bearing mice were enlarged 2 weeks after tumour injection; most of the splenocytes were large null cells outside the size range of lymphocytes. Furthermore, of those cells within lymphocyte size range, ~45% were null-staining cells. We do not know the origin or function of these cells but in view of their prevalence we are currently trying to determine their significance.

Although this work was performed using the C57BL/6, EL-4 model, we have noted similar thymic regression in many other host–tumour combinations and have confirmed the selection of mature thymocytes by FACS analysis in DBA/2 mice given the L5178Y tumour.

We wish to thank Professor L. A. Herzenberg in whose laboratory the work was performed and Dr J. Ledbetter who kindly donated the monoclonal reagents. The work was partly supported by the Cancer Research Campaign.

REFERENCES

Aoki, T. & Johnson, P. A. (1972) Suppression of gross leukaemia cell surface antigens: A kind of antigenic modulation. *J. Natl Cancer Inst.*, **49**, 183.

Apffel, C. A., Arnason, B. G., Twinam, C. W. & Harris, C. A. (1966) Recovery with immunity after serial tapping of transplantable mouse ascites tumours. *Br. J. Cancer.* **20**, 122.

Berendt, M. J. & North, R. J. (1980) T cell mediated suppression of anti-tumour immunity: An explanation for progressive growth of an immunogenic tumour. *J. Exp. Med.*, **151**, 69.

Bertschmann, M., Scharen, B. & Lusher, E. F. (1979) Correlation of *in vivo* and *in vitro* immune reactions against intradermally developing P-815 mastocytoma in the syngeneic mouse. *Immunobiology*, **156**, 382.

Chassoux, D., McLennan, I. C. M. & Munro, T. R. (1977) Competition for cytotoxic immune capacity against a syngeneic mouse tumour distributed at two sites. *Int. J. Cancer*, **19**, 796.

Eckhardt, L. A. & Herzenberg, L. A. (1980). Monoclonal antibodies to ThB detect close linkage of Ly-6 and a gene regulating ThB expression. *Immunogenetics*, **11**, 275.

Gershon, R. K., Mokyr, M. B. & Mitchell, M. S. (1974) Activation of suppressor T cells by tumour cells and specific antibody. *Nature*, **250**, 594.

Hellström, K. E. & Hellström, I. (1974) Lymphocyte mediated cytotoxicity and blocking serum activity to tumour antigens. *Adv. Immunol.*, **18**, 209.

Kemp, A., Berke, G., Crowell, J. & Amos, B. (1973) Induction of cell mediated immunity against leukemia EL4 in C57BL mice. *J. Natl Cancer Inst.*, **51**, 1877.

Kuperman, O., Fortner, W. & Lucas, Z. (1975) Immune response to a syngeneic mammary adenocarcinoma. III. Development of memory and suppressor functions modulating cellular cytotoxicity. *J. Immunol.*, **115**, 1282.

Ledbetter, J. A. & Herzenberg, L. A. (1979) Xenogeneic monoclonal antibodies to mouse lymphoid differentiation antigens. *Immunol. Rev.*, **47**, 63.

LEDBETTER, J. A., ROUSE, R. V., MICKLEM, H. S. & HERZENBERG, L. A. (1980) T cell subsets defined by expression of Lyt-1, 2, 3 and Thy-1 antigens: Two parameter immunofluorescence and cytotoxicity and analysis with monoclonal antibodies modifies current views. *J. Exp. Med.*, **152**, 280.

MICKLEM, H. S. LEDBETTER, J. A., ECKHARDT, L. A. & HERZENBERG, L. A. (1980) Analysis of lymphocyte subpopulations with monoclonal antibodies to Thy-1, Lyt-1, Lyt-2 and ThB antigens. In *Regulatory T Lymphocytes* (Eds. Pernis & Vogel). New York: Academic Press. p. 119.

OI, V. T. & HERZENBERG, L. A. (1979) Localisation of murine Ig-1b and Ig-1a ($IgG_{2a}$) allotypic determinants detected with monoclonal antibodies. *Molec. Immunol.*, **16**, 1005.

PERRY, L. L., BENACERRAF, B. & GREENE, M. I. (1978) Regulation of the immune response to tumour antigens, IV. Tumour antigen-specific suppressor factor(s) bear I-J determinants and induce suppressor T cells *in vivo*. *J. Immunol.*, **121**, 2144.

ROGERS, P. & MATOSSIAN-ROGERS, A. (1981) Selection of thymocytes with phenotypes of mature T cells using corticosteroids. *I.R.C.S. Med. Sci.*, **9**, 564.

ROGERS, P. & MATOSSIAN-ROGERS, A. (1982) Differential sensitivity of lymphocyte subsets to corticosteroid treatment. *Immunology*, (in press).

SJÖGREN, H. O., HELLSTRÖM, I., BANSAL, S. C. & HELLSTRÖM, K. E. (1973) Suggestive evidence that the blocking antibodies of tumour bearing individuals may be antigen–antibody complexes. *Proc. Natl Acad. Sci.*, **68**, 1372.

TAKEI, F., LEVY, J. G. & KILBURN, D. K. (1977) Characterisation of suppressor cells in mice bearing syngeneic mastocytoma. *J. Immunol.*, **118**, 412.

WAGNER, H., HARDT, C., BARTLETT, R., ROLLINGHOFF, M. & PFIZENMAIER, K. (1980) Intrathymic differentiation of cytotoxic T lymphocyte (CTL) precursors. I. The CTL immunocompetence of peanut agglutinin-positive (cortical) and negative (medullary) Lyt 123 thymocytes. *J. Immunol.*, **125**, 2532.

WHITNEY, R. B. & LEVY, J. G. (1975) Effects of sera from tumour-bearing mice on mitogen and allogeneic cell stimulation of normal lymphoid cells. *J. Natl Cancer Inst.*, **45**, 733.